# On the Use of a Simple Physical System Analogy to Study Robustness Features in Animal Sciences

**DOI:** 10.1371/journal.pone.0137333

**Published:** 2015-08-31

**Authors:** Bastien Sadoul, Olivier Martin, Patrick Prunet, Nicolas C. Friggens

**Affiliations:** 1 INRA, LPGP Fish Physiology and Genomics UR1037, Rennes, France; 2 INRA, Modélisation Systémique Appliquée aux Ruminants UMR0791, Paris, France; 3 AgroParisTech, Modélisation Systémique Appliquée aux Ruminants UMR0791, Paris, France; Indian Institute of Toxicology Reserach, INDIA

## Abstract

Environmental perturbations can affect the health, welfare, and fitness of animals. Being able to characterize and phenotype adaptive capacity is therefore of growing scientific concern in animal ecology and in animal production sciences. Terms borrowed from physics are commonly used to describe adaptive responses of animals facing an environmental perturbation, but no quantitative characterization of these responses has been made. Modeling the dynamic responses to an acute challenge was used in this study to facilitate the characterization of adaptive capacity and therefore robustness. A simple model based on a spring and damper was developed to simulate the dynamic responses of animals facing an acute challenge. The parameters characterizing the spring and the damper can be interpreted in terms of stiffness and resistance to the change of the system. The model was tested on physiological and behavioral responses of rainbow trout facing an acute confinement challenge. The model has proven to properly fit the different responses measured in this study and to quantitatively describe the different temporal patterns for each statistical individual in the study. It provides therefore a new way to explicitly describe, analyze and compare responses of individuals facing an acute perturbation. This study suggests that such physical models may be usefully applied to characterize robustness in many other biological systems.

## Introduction

The ability of an organism to respond to an environmental perturbation is of growing scientific concern in animal ecology and in animal production sciences [1–3]. Indeed, in the light of global climate change, the number and the suddenness of extreme meteorological events like drought, flood and storms is increasing. In parallel, despite controlled rearing conditions, environmental perturbations are frequent in animal production and can appear in an unpredictable manner. Depending on animal vulnerability, these environmental perturbations can affect the health, welfare, and fitness of the animal [4]. In this context, a significant effort is being devoted characterization of robust animals. However, the robustness of biological systems is known to be a complex feature difficult to access and characterize [5,6]. One major component of robustness is the capacity to cope with environmental perturbations, typically via elasticity in sub-elements of the system. Recent work suggests that quantifying robustness needs a multivariate approach and that key information about the ability of animals to face acute perturbations can be derived from the dynamic of responses to a perturbation [7,8]. The above studies have provided useful statistical tools for doing this and gaining some insight into the underlying biological processes. However, they do not per se attempt to describe basic features of the adaptive system. If response-recovery dynamics could be quantified in terms of generic components, this may facilitate a better characterization of adaptive capacity, and thereby robustness.

Physics and biology share many similarities, starting with adjectives and concepts [9]. Terms like plasticity, resistance or elasticity are, indeed, all borrowed from physics and allow the description of some robustness features of biological systems facing environmental perturbations. Thus, a robust biological system is, for some authors, expected to be resistant to acute environmental perturbations, and to show good recovery capacities [3,10,11]. Taking this one step further, it seems likely that the use of an interdisciplinary approach, combining physics and animal science, might help to characterize some generic features of the complex trait of robustness. Thus, we propose in this study that biological systems facing an acute perturbation can be described in terms of resistance and recovery abilities using the simple physics model of a spring and a damper. Similar models were previously developed for describing aspects of locomotion in human (see for review, [12] or for explaining complex hormonal oscillatory mechanisms [13] but never to characterize robustness features in the animal sciences. Therefore, this study aims to (1) investigate the suitability of this model to describe the responses of animals to perturbations, and (2) show a concrete example of how this model can be applied in practice in studies investigating the effect of a perturbation on animals. This second point required data on individuals facing an acute perturbation that were monitored before, during and after the perturbation. Data from a published study investigating the effect of a confinement perturbation on fish were used [8].

## Materials and Methods

### General approach

**Fig 1:**
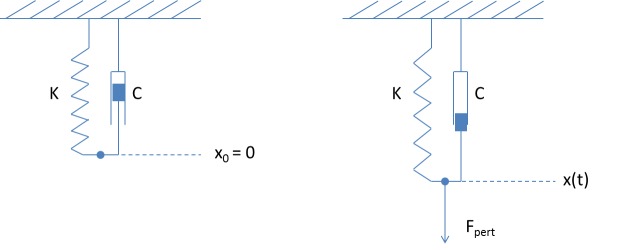
The Kelvin-Voigt model in a non-perturbed environment (1.a) and during a perturbation (1.b). The model is composed of a spring and a damper in parallel and characterized respectively by the parameters K and C. During a perturbation a force of perturbation (F_pert_) pulls on the system and the measure of interest, x(t), is increased.

The model proposed in this study is mathematically formulated in terms of a generic model in which a spring and a damper are set in parallel, a model also called the Kelvin-Voigt model [14]. The deformation of the model is given by the x coordinates of the end-point (Fig 1). This model is composed of 2 main parameters describing the properties of the spring and damper (K and C). An environmental perturbation is represented as a force (F_pert_) that pulls the spring and damper system. We assume that before the challenge, the system is in a non-challenging environment where the force on the model is zero. Because animals might perceive differently a challenge that is physically the same, we envisage the modulation of the force of the perturbation F_pert_ by a coefficient of perception (perc; range 0 to 1).

### Model formalism

The system follows the differential Eq 1.1, determined using the second rule of Newton, where *a* is the acceleration (i.e. d^2^x/dt) and *v* the velocity (i.e. dx/dt) of the end-point of coordinate *x*. During the pre-challenge period (t< τ_1_), the environment is considered as not affecting the system. The same assumption is made for the period after the challenge (t ≥ τ_2_). The term percF_pert_ is therefore considered as negligible during these periods, leading to the Eq 1.2. When the perturbation occurs (τ_1_ ≤ t < τ_2_), the system is affected by a non-negligible environmental perturbation and Eq 1.1 can be rearranged to Eq 1.3.

$$percF_{pert}\;*\;a(t)=\;percF_{pert}-Kx(t)-Cv(t)$$ (1.1)

$$v(t)=-\frac{K}{C}\;x(t)\;t<\tau_{1}\;or\;t\geq \;\tau_{2}$$ (1.2)

$$a(t)=\frac{1}{percF_{pert}}\left(\;percF_{pert}-Kx(t)-Cv(t)\right)\;\tau_{1}\leq t<\;\tau_{2}$$ (1.3)

**Fig 2:**
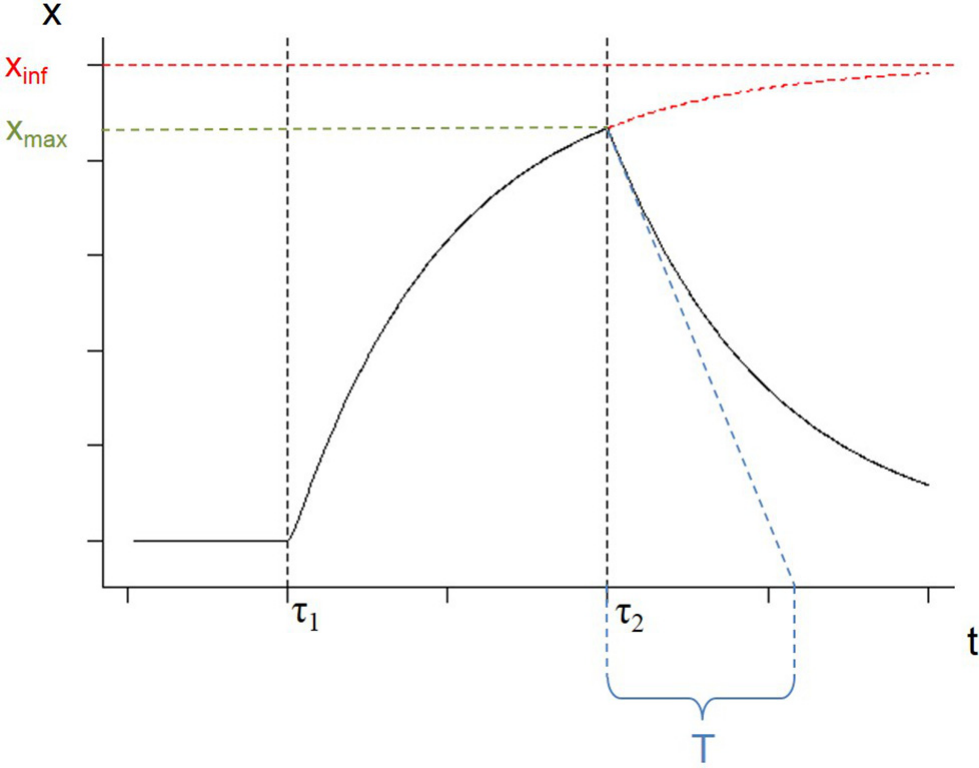
An example of the outcomes of the model and its extrapolated features when continuously and constantly perturbed between τ_1_ and τ_2_. We define the value T = C/K as the decay constant, characterizing the recovery capacity of the system. The value x_inf_ corresponds to the asymptotic value of x if the perturbation continues indefinitely, whereas x_max_ is defined as the value of x at the end of a time-limited perturbation.

If the challenge was to persist, for *t* → +∞, because a and v would tend towards 0, we obtain from Eq 1.3 the asymptotic elastic solution $x_{inf}=\frac{percF_{pert}}{k}$ (Fig 2). During the recovery (t ≥ τ_2_), we define the value T = C/K as the decay constant, characterizing the recovery capacity of the system (Fig 2) and x_max_ the value of x at the end of the perturbation.

### Sensitivity analysis

In order to describe the flexibility of the model, an analysis of sensitivity was performed by varying one of the parameters K, C or F_pert_, fixing arbitrarily the two others and running the model. The range of variation of the studied parameter and the values of the fixed parameters were chosen in order to create figures that clearly illustrate the impact of the variation in the studied parameter. We considered a situation where x is followed during 1000 iterations represented on a time scale of 100, i.e. with a time step of 0.1 time units, and where a constant perturbation F_pert_ occurs between time 20 and 60.

First, values of K were chosen between 0.1 to 1, whilst C and F_pert_ were set to 2 and 1 respectively. Secondly, values of C varied between 0.5 to 20, whilst K and F_pert_ were set to 0.1 and 1 respectively. Finally, values of Fpert varied between 1 and 10, whilst K and C were set to 0.1 and 2 respectively. For each analysis of sensitivity, the effect of the changing parameter on x_max_, x_inf_ and T was shown in a continuous graphic.

### Application of the model to a study on the effect of a perturbation in rainbow trout

Data of a study previously published [8] were used in this analysis. These are briefly summarized here, for further details please refer to Sadoul et al [8].

**Fig 3:**
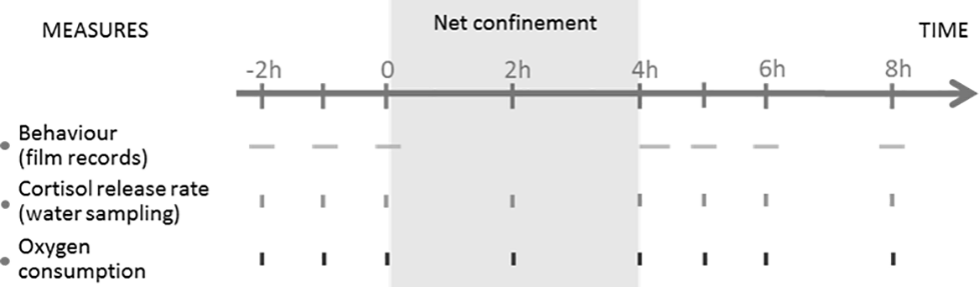
Design of the confinement experiment during time. Aquaria are followed during time for behavior, cortisol release rate and oxygen consumption. Each horizontal line represents a 10 minutes film record taken by camera placed above the aquaria. Vertical lines correspond to the water sampling points and the oxygen consumption measurements. At time 0, a 4-hour confinement challenge is performed by enclosing all the fish of one aquarium in a net, creating a density of 140kg/m3.

Eight aquaria of 1.7L containing each 16 juvenile rainbow trout of similar size were used. Half of the aquaria contained only fish from an isogenic line A22-B57 (called A), the other half contained fish from an isogenic line R23-B57. Cortisol release rate, oxygen consumption and group behavior (group dispersion and group activity) were followed at different time points distributed within 10 hours (Fig 3). A confinement challenge of 4 hours was performed on all aquaria by grouping all the fish of one aquarium in a net. The density was therefore suddenly increased up to 140 kg/m^3^ creating a stressful situation for the fish [15–17].

The experiment was carried out in two replicates one month apart with fish from the same fecundation. Within each replicate, both lines were used. Therefore, for each line a total of 8 aquaria was used.

### Data preparation

The data preparation steps were based on two hypotheses consisting of (1) considering that the unit of the measures do not have an impact on the parameters of the model (e.g. an increase of 1000 is not more important that an increase of 100 if the initial values are respectively 1000 and 100), and (2) the individuals in the aquarium were, at least at one time point, close to a non-disturbed state (generally just before the challenge or at the end of the recovery period).

Two data preparation steps were conducted to fit to these two hypotheses. In the first step, each measure of each individual was normalized using the mean of all aquarium of the corresponding measure, creating a fold change of the mean measure. The second step consisted in subtracting, for each individual and measure, the minimum of the normalized individual temporal pattern. All the temporal patterns were therefore shifted so that the minimum value was set to 0.

The 10 hours of the experiment were transformed onto a 0 to 100 scale, with the challenge occurring between 20 and 60. This arbitrary scale from 0 to 100 will help comparison with future analyses initially on different time scales.

### Model assumptions and model fitting

The perturbation force F_pert_ was assumed to be positive, creating an extension of the spring. During the perturbation, x(t) will therefore increase and the variations in the different measures need to be positive. In this study we assume that all aquarium are subjected to the same perturbation (F_pert_), arbitrarily set to 0.1. For simplicity, the differences between aquaria in perception of the perturbation (perc) are assumed to be negligible here, and therefore, the perception is set to 1 for all individuals. In this study, fitting the model consists therefore in optimizing only the parameters K and C of the model. The system was described to be in its non-disturbed state when x = 0. The optimization procedure was performed using the *optim* function on differential Eqs 1.2 and 1.3 solved using the *ode* function in the free software R 2.14.0 (http://cran.r-project.org/). The code of the model and an example of use is given in S1 Appendix for a use with the software R.

The optimization procedure was first run on the mean temporal pattern of each measure, to assess the ability of the model to fit different experimental data.

The model was then optimized on each individual temporal pattern for each measure, providing a K_i,m_, and a C_i,m_ for individual i and measure m.

### Model outputs analysis

Model fitting performance was calculated for each aquarium and each measure using the modeling efficiency statistic (MEF) as described in the review of Tedeschi [18] and calculated using the following formula, with *Y*_*i*_ the observed value i, *Ymod*_*i*_ the corresponding model value and $\bar{Y}$ the observed mean:

$$MEF=1-\frac{\sum_{i}{\left(Y_{i}-{Ymod}_{i}\right)}^{2}}{\sum_{i}{\left(Y_{i}-\bar{Y}\right)}^{2}}$$

A MEF value of one would indicate a perfect fit. On the contrary, a negative value of MEF suggests that the model values are worse than the observed mean.

For the purpose of the group level statistics, the few results where the model was not able to correctly fit the individual temporal pattern (when C or K is outwith ± 1.5 times the interquartile range) were withdrawn from the analysis. This corresponded to 1 aquarium (line A) for the cortisol release rate, 3 (2 line A, 1 line R) for the oxygen consumption, 0 for the group activity and 3 (1 line A, 2 line R) for the dispersion.

For statistical analyses, Ks and Cs were log-transformed to increase normality. For each measure, the effect of line and replicate on the corresponding K and C were assessed using a linear mixed model fitting each parameter with line, replicate and the interaction as fixed effects, and aquarium as a random effect. Non-significant fixed effects were withdrawn from the model and the model was re-run. The mean estimation of K and C for each line and the p-value for the line effect were extracted from the final model. Correlations between parameters were calculated using simple Pearson’s correlation coefficients.

All statistical analyses were run using the free software R 2.14.0 (http://cran.r-project.org/).

## Results

### Model flexibility

**Fig 4:**
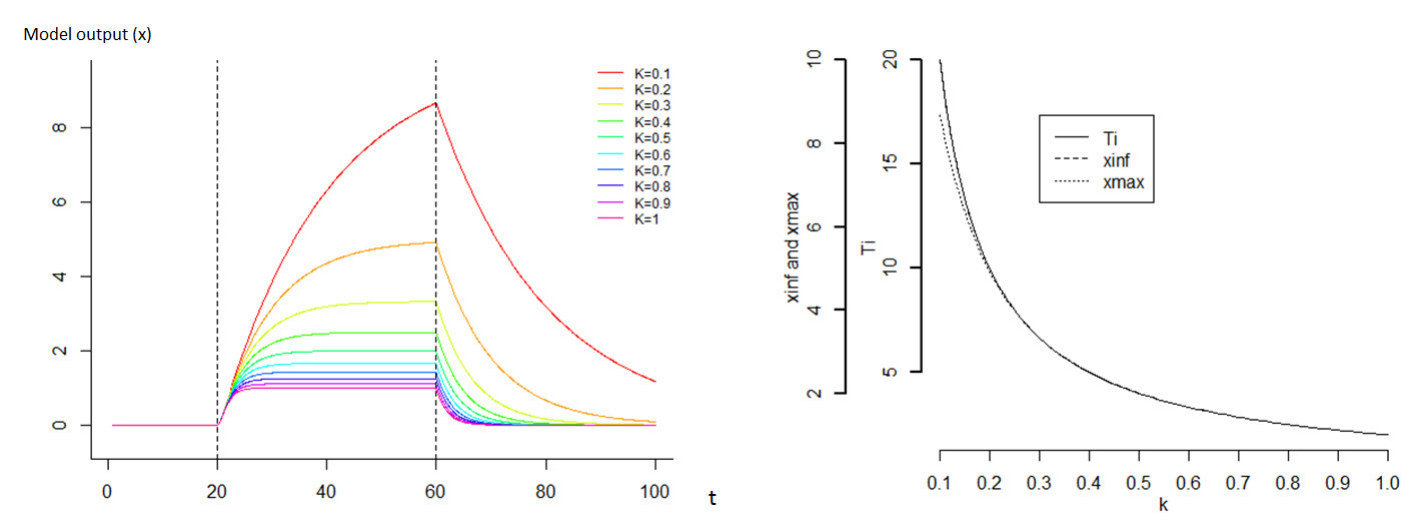
Sensitivity analysis of the model where the parameter K varies and a continuous perturbation occurs between time 20 and 60. For this analysis, perc was set to 1, Fpert to 1 and C to 2. K varied between 0.1 and 1. We define the value T = C/K as the decay constant, characterizing the recovery capacity of the system, x_max_ the value of x at the end of the perturbation and x_inf_ the value of x if the perturbation continues indefinitely.

**Fig 5:**
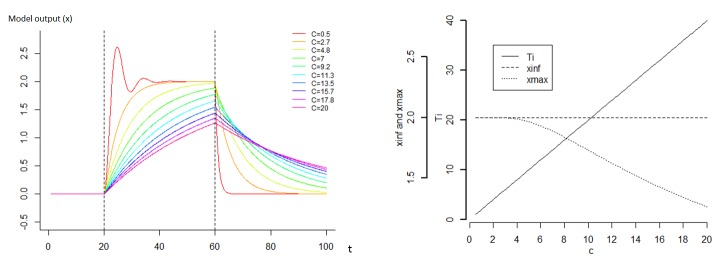
Sensitivity analysis of the model where the parameter C varies and a continuous perturbation occurs between time 20 and 60. For this analysis, perc was set to 1, K to 0.1 and Fpert to 1. C varies between 0.5 and 20. We define the value T = C/K as the decay constant, characterizing the recovery capacity of the system, x_max_ the value of x at the end of the perturbation and x_inf_ the value of x if the perturbation continues indefinitely.

**Fig 6:**
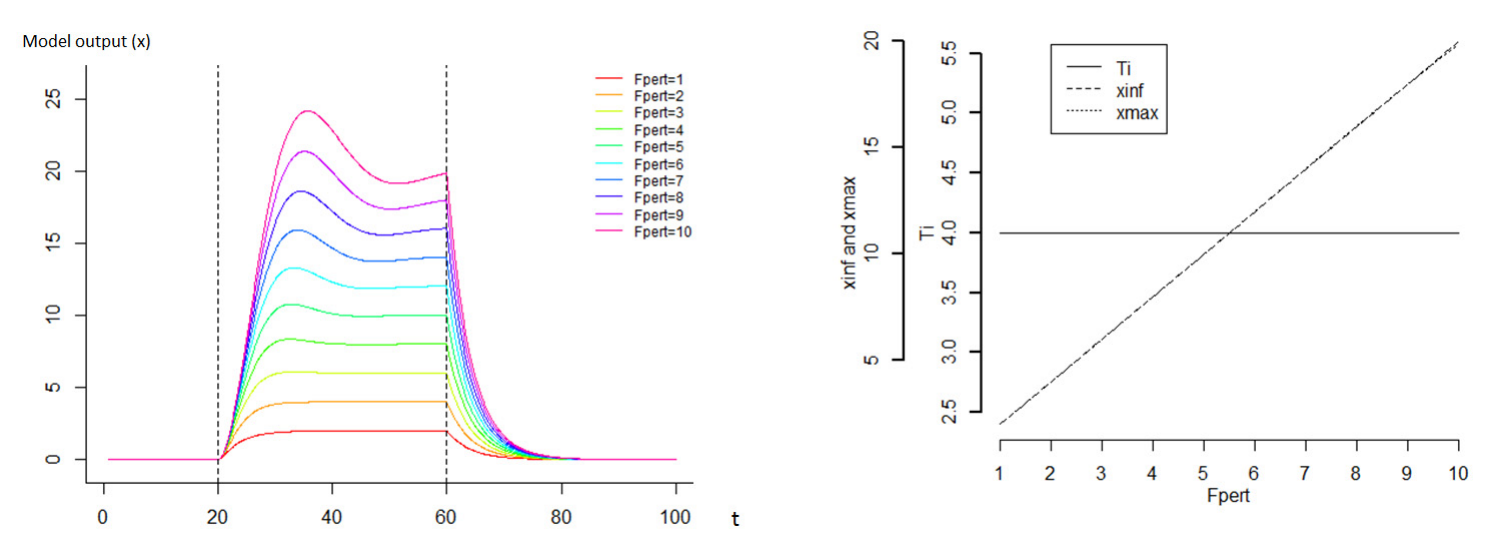
Sensitivity analysis of the model where the parameter Fpert varies and a continuous perturbation occurs between time 20 and 60. For this analysis, perc was set to 1, K to 0.1 and C to 2. F_pert_ varies between 1 and 10. We define the value T = C/K as the decay constant, characterizing the recovery capacity of the system, x_max_ the value of x at the end of the perturbation and x_inf_ the value of x if the perturbation continues.

**Fig 7:**
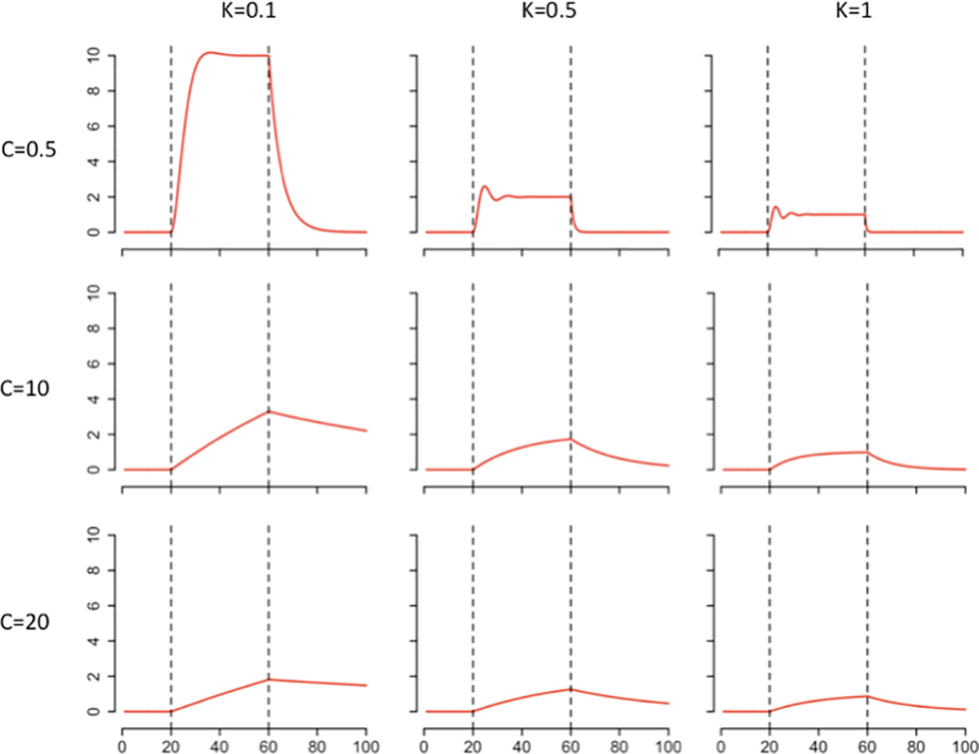
Simulated curves showing the interplay between K and C in a sensitivity analysis of the model where a continuous perturbation occurs between time 20 and 60. K and C take each three different (low, mid, high) values.

The sensitivity analysis showed that varying C and K in the model resulted in a strong diversity of dynamics (Figs 4 and 5). As expected, the parameter K has a strong effect on the amplitude of the response (Fig 4). The relationship between the maximum of the amplitude and K is approximately hyperbolic, with low values of K strongly increasing the amplitude of the response. Similarly, the time of recovery T is approximately hyperbolic to parameter K, with a strong decrease when K increases. Globally, parameter C has a strong impact on the shape of the response since it influences the speed of the deformation (Fig 5). This parameter has no impact on the asymptotic x (x_inf_), however it impacts the x_max_ value if the time of perturbation is not long enough to enable the system to reach x_inf_. When the time of perturbation is low, the value x_max_ and C are negatively correlated. The relationship between the time of recovery and C is proportional. The variable F_pert_ has a positive linear impact on the amplitude of response x_max_ and x_inf_ (Fig 6). However, it has no impact on the recovery. The effect of K on the response is highly dependent on C (Fig 7). Globally, K has a stronger impact on the amplitude of the response when C is low. The parameter K defines therefore the asymptote value that a given perturbation would produce if it continued for long enough. We call K the “deformation potential” or the stiffness of the system. On the contrary, C has a strong effect on the shape of the response. The C effect is less impacted by the value of K, and can be defined as the “capacity to impede the deformation” or the resistance of the system to the deformation.

### Adjustment to mean responses in a real study

**Fig 8:**
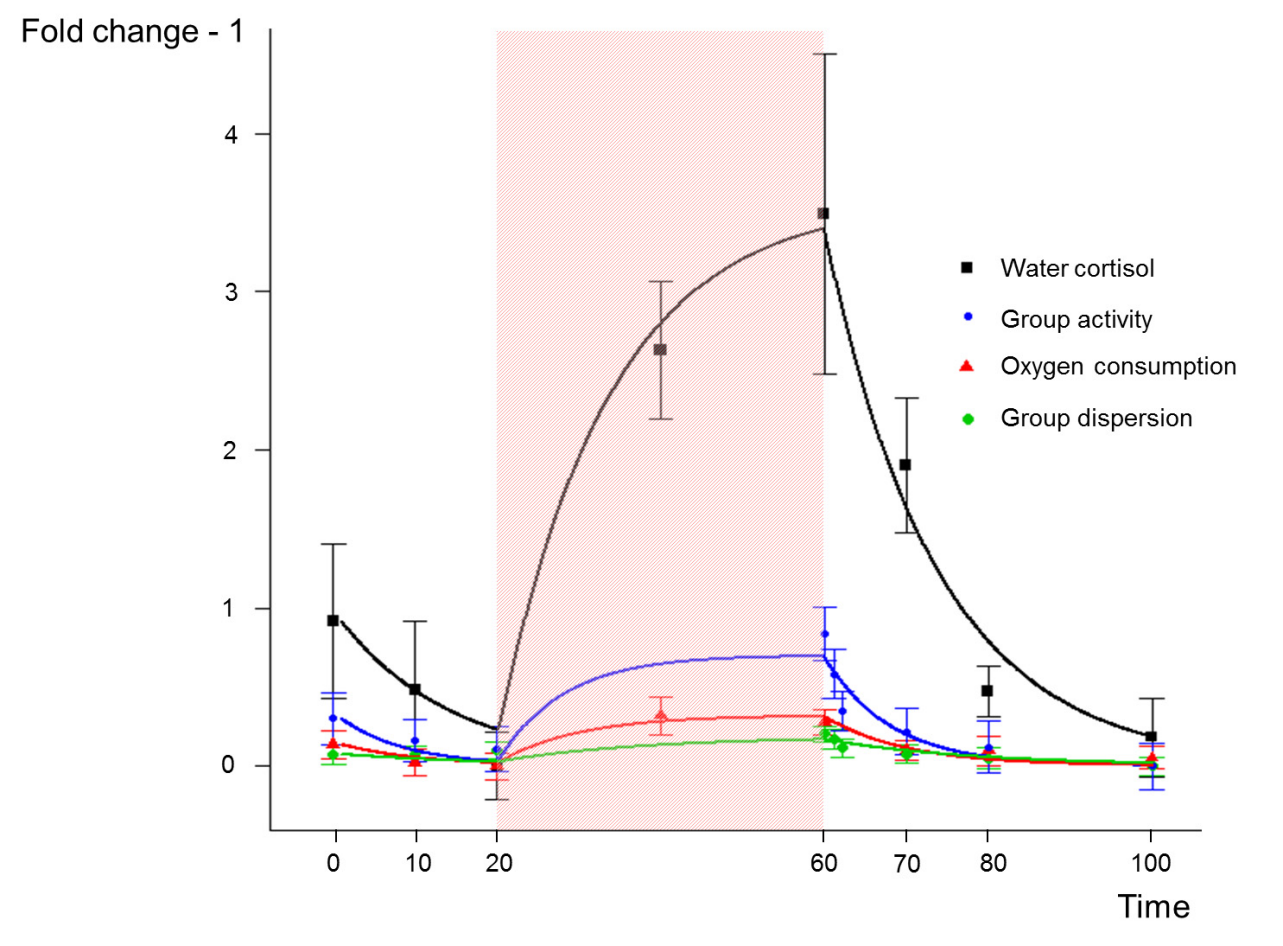
Fitting the model to cortisol release rate, oxygen consumption, group activity and group dispersion in rainbow trouts facing and recovering from a confinement challenge (grey rectangle). Data (observed values ± SE) are expressed as fold changes. The model fit is shown for each measure by the solid line. Stiffness (K) and resistance to change (C) parameters of the model were fitted on the mean of the cortisol release rate (K = 0.028, C = 0.384), group activity (K = 0.143, C = 1.137), oxygen consumption (K = 0.314, C = 3.052) and the group dispersion (K = 0.542, C = 9.809) measures.

After data preparation (see dedicated section in Materials and Methods), the mean temporal pattern for each measure was calculated and presented, as points, in Fig 8. Globally, fish subjected to a confinement challenge display an increase of cortisol release rate, oxygen consumption and group behavior (activity or dispersion). The model was adjusted to the four mean responses to evaluate its ability to fit different physiological and behavioral responses to a perturbation. The adjustments were, despite the amplitude differences, very satisfactory (continuous lines in Fig 8) with high model efficiency statistics (MEF = 0.98, 0.88, 0.90, 0.67 for the cortisol release rate, the group activity, the oxygen consumption and the group dispersion, respectively). The highest model values were obtained at the end of the confinement period. The 4 measures show very divergent amplitudes of response to the challenge, translated by contrasted K values. Cortisol release rate increased 4-fold relative to baseline values (x_max_ = 4.4). In contrast, group dispersion increased only 1.3 fold (x_max_ = 1.31). These differences in amplitude result in a stiffness 11 times (K_disp_/ K_cort_) stronger for group dispersion than for cortisol (Table 1).

**Table 1:** Model estimated (K and C) and derived (T and xinf) parameters, for two isogenic lines subjected to a confinement challenge.

	Cortisol release rate				Oxygen consumption				Group dispersion				Group activity			
	K	C	T	x_inf_	K	C	T	x_inf_	K	C	T	x_inf_	K	C	T	x_inf_
Line A	0.018	0.302	17.501	6.628	0.080	1.074	17.825	1.312	0.453	3.871	18.814	0.26	0.120	0.740	9.275	1.022
Line R	0.039	0.537	14.799	2.808	0.179	1.449	11.889	0.724	0.245	5.791	41.657	0.45	0.126	2.428	25.539	0.920
p-value	0.003	0.051	0.260	<0.0001	0.089	0.526	0.270	0.016	0.058	0.609	0.174	0.028	0.8398	0.075	0.027	0.733

The significance of the line effect is given by the p-value extracted from a linear mixed model where K or C is explained by line and, if significant, replicate and interaction, including aquarium as a random effect.

### Adjustment to individual responses

The induced stress responses were adjusted using the model for each variable and each individual aquarium, producing one K and C for each. The results were summarized in Table 1 presenting the average K and C for each line and each measure, and the significance of the line effect on the parameters. The cortisol release data show a significant difference in K, with line R being two times stiffer than line A. Similarly, line R had an almost significantly (p-value = 0.051) greater C value than line A. In consequence, the two lines show very similar recovery rates (T) for the cortisol release rate. For the oxygen consumption, line R tends (p-value = 0.08) to show a stronger K than line A. This is translated by the significantly greater x_inf_ value for line A. In contrast, line A showed a stronger stiffness in the group dispersion measure (p-value<0.1) with a significantly lower x_inf_. For group activity, the two lines do not differ for the parameter K but a significant difference in the time of recovery (T) was found, due to the C parameter.

### Between parameter correlations

The correlations between the different K and C for each measure are shown in Table 2. Within measure K and C were positively correlated for all measures (except for group dispersion, p-value = 0.13). In addition, Ks of the two physiological parameters (water cortisol and oxygen consumption) were positively correlated. A similar trend was found between (p-value = 0.056) the Ks of the two behavioral parameters. Similar results were found for the Cs. The parameter C of the cortisol release rate tended to be negatively correlated with the behavioral Cs.

**Table 2:** Pearson’s correlation coefficients between model estimated parameters.

		Cortisol release rate		Group dispersion		Oxygen consumption		Group activity	
		K	C	K	C	K	C	K	C
Cortisol release rate	K	1	0.94***	-0.17	-0.42	0.56*	0.36	-0.30	-0.31
	C		1.00	-0.16	-0.42	**0.51°**	**0.43°**	-0.39	**-0.47°**
Group dispersion	K				0.39	0.43	0.07	**0.49°**	0.11
	C				1.00	0.13	-0.22	0.33	0.52*
Oxygen consumption	K					1.00	0.61*	0.27	0.08
	C						1.00	-0.18	-0.15
Group activity	K							1.00	0.54*
	C								1.00

Significant correlations or tendencies (P<0.1) shown in bold (***: P<0.001, *: P< 0.05 and °: P<0.1).

## Discussion

The purpose of this study was to see if a simple physics model of elasticity could be useful for describing an important diversity of temporal dynamics during and after an environmental perturbation of a biological system. Fitting the model to experimental data on multiple measures of stress in fish subjected to an acute perturbation demonstrated the capacity of the model to characterize the resistance and the ability to recover from a perturbation for each measure.

### The Model parameters

The environmental perturbation, by “pulling” on the system, creates a “distortion” and changes the values of the system measure. The temporal patterns of the four responses (cortisol release, O2 consumption, dispersion, and activity) were found to fit the dynamic equation of a spring and a damper. The parameter describing the stiffness of the system (K) provides an explicit measure of the deformation potential of the biological system when facing a given perturbation. The Ks can be compared between responses and individuals to describe which one can be the most modified by the perturbation. On the other hand, the parameter C of the equation, corresponding to the friction of the damper, can be considered as a measure of the capacity of the biological system to resist a change. With a similar K, a low C translates a rapid change of the system response. Rigidity, defined as a small response of the system to the perturbation, can be obtained using two different strategies when facing an acute perturbation, either by showing a strong K, reducing the potential of deformation of the system, or by showing a strong C, impeding the deformation. Therefore, during an acute perturbation, a strong K or a strong C lead to a good rigidity of the system (see Fig 7). These two strategies are probably the results of different underlying biological mechanisms.

The combination of the two parameters also enables the calculation of the recovery capacity of the system (T). This capacity is strong when C is low and K is high. Therefore, by considering the system using a physics model, important features of the system; resistance, rigidity and recovery capacity, can be explicitly described.

In animal science and ecology, resistance is considered to be an important ability of the animal. Resistance allows the animal to minimize changes in life functions that are important for productivity or reproduction when facing acute environmental perturbations [2,3]. Similarly, an animal taking short period of time to recover from a disturbance is of great interest since it can return quickly to its target levels of functioning [10,19,20]. However, the extent to which resistance is advantage or disadvantage for the animal depends on the nature of the perturbation. Given that, in our confinement experiment the perturbation used is not life-threatening, since it does not affect water quality or fish integrity, the individuals showing strong resistance and good recovery capacities are the individuals that avoid triggering unnecessary physiological and behavioral responses. In this experiment, we can therefore define individuals having strong resistance and good recovery capacities as robust animals [3].

### Physiological and behavioral dynamics analyzed using the model

All of the measures obtained from fish subjected to a confinement showed an increase during the perturbation compared to before. These variations were detectable and quantifiable using the present model. The model was found to adequately fit the data at the level of the individual, and therefore seems to be a relevant method for describing the responses of perturbed systems. In contrast to a previous analysis of these data [8], where the different periods of the measurement profiles (increase and decrease) were analyzed separately and sequentially, the use of the present model allowed us to analyze simultaneously all points of the temporal patterns and reduce therefore the effect of unexplained variance of isolated time points. Furthermore, the model provides comparable data in standardized common units facilitating between-challenge interpretation, between-response correlations or between-subject comparisons. Being able to describe temporal patterns in a way that allows inference across measures, or between individuals is often an important challenge in experimental studies [7,21–23].

The present model describes an increase and an early attainment of values close to the maximum response in group behavior during the challenge. Interestingly, although these dispersion and activity behaviors cannot be directly measured during the challenge–because it is a confinement challenge- the model provides a prediction of how they would evolve had the fish been able to express these behaviours. Even if in this experiment there was no way of testing the validity of these predictions, it remains an interesting property of the model to be able to intuit the motivation to express a behaviour [24].

With respect to the physical measures, oxygen consumption and cortisol release, line R showed stronger resistance (stronger K and stronger or similar C) to the challenge compared to line A. The time to recover from the challenge was similar between the two lines for these physiological measures. These results confirm the findings in a previous analysis of the data [8]. However, the present analysis showed divergent group behavior responses between the two lines that were not detected in the previous analysis. Indeed, line A showed stronger stiffness to the challenge when considering the group dispersion behavior, and better recovery ability when considering the group activity behavior. Thus, these results indicate the existence of divergent coping strategies between the two lines, with one line being more sensitive in its physiological mechanisms and one line being more affected in its behavioral parameters.

### Model implications and potentials

The present model was constructed to be sufficiently generic to be able to describe very different responses. We have shown in this study its ability to fit with physiological and behavioral stress related measures. By using a common model across measures, we can extract from diverse temporal patterns with various scale units, comparable features of resistance and recovery capacities. Given this, it seems likely that such a model could help characterize perturbations of various types, e.g. social stress versus physical stress, and compare them on a same common scale. This model would, for example, have been of particular use for estimating the difference of perturbation intensity (F_pert_ in our model) in welfare studies evaluating stress dynamics (sensitivity and recovery) depending on different intervention methods (see for review [25]).

**Fig 9:**
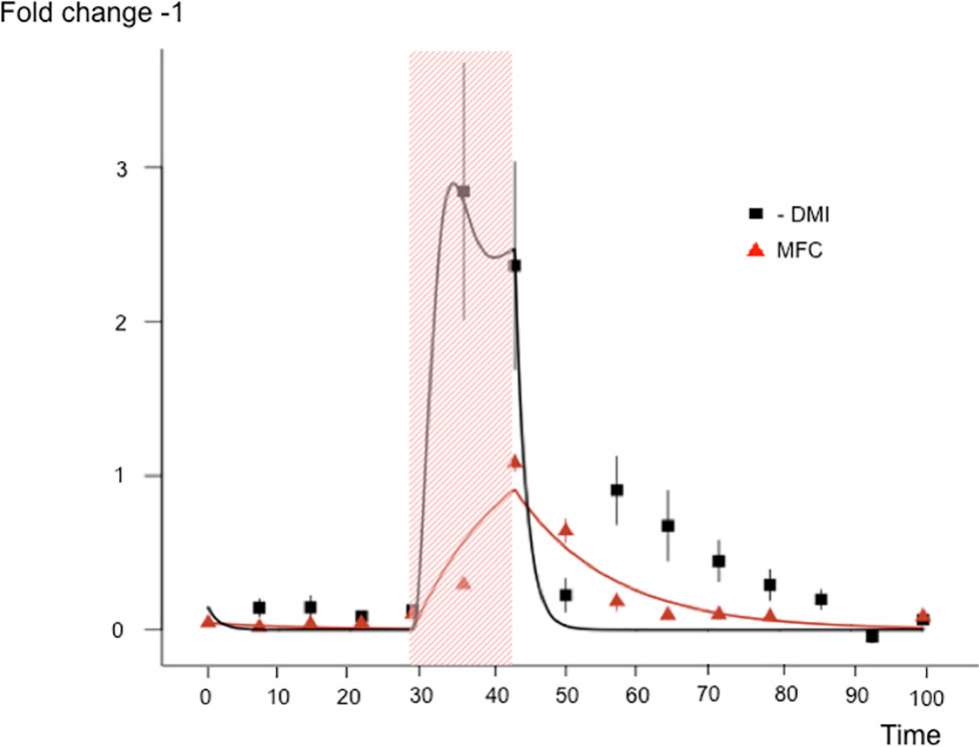
Fitting the model to Dry Matter Intake (DMI) and Milk Fat Content (MFC) in goats before, during and after a 2 days nutritional challenge (grey rectangle). Data (observed values ± SE) are expressed as fold changes. The inverse of DMI is illustrated since DMI is decreasing during the challenge. Stiffness (K) and resistance to change (C) parameters of the model were fitted on the mean of the DMI (K = 0.04, C = 0.06) and MFC (K = 0.72, C = 0.97) measures.

To illustrate its genericity, the model was tested on data obtained from a different type of challenge, in a different species; a nutritional challenge in goats [26]. In this study, 16 lactating goats were followed every day for dry matter intake (DMI) and milk fat content (MFC). Goats initially fed a standard diet were suddenly subjected to a 100% straw diet during 2 days. The measures were recorded before, during and after the nutritional challenge. The present model was fitted to MFC and DMI measures as described in the Materials and Methods. Fig 9 shows that the model was able to correctly fit the responses to the challenge providing values of resistance and recovery for MFC. However, the fitting of the recovery period for DMI was not totally satisfactory since the temporal pattern of DMI seems to display a rebound phenomenon that the model cannot capture in its present form. This type of phenomenon has been observed in other measures [23,27] and could be captured by adapting the damped spring model to include a mass during the recovery phase. However, this and the possible elaborations of the model would require further work, to explore the conceptual value of such modifications.

In addition to using the model to characterize planned challenges, we can also envisage using the model for extracting and analyzing the perturbation dynamics due to unplanned micro-environmental variations in natural or rearing conditions. Indeed, new technologies based on video analysis or biosensors are rapidly developing and are increasingly being deployed to monitor domestic or wild animals. These can produce large amounts of dynamic data on behavioral and physiological stress mechanisms, which require new tools to analyze and interpret [8,28–30]. Recent work described a method to identify periods of deviance from the basic performance trajectory of cows monitored for their milk yield [31]. Combining this method and our model could potentially enable the identification and characterization of resistance and recovery capacities from dynamic data of monitored animals under natural conditions or in normal rearing systems. This would be an important step towards large scale quantification of the adaptive capacity of animals under normal applied conditions. The basic model presented in this study could be extended to be able to accommodate other types of perturbation, e.g. chronic, combined or repeated perturbations. Indeed, by adding some specific features to the model, an updated model could potentially accommodate notions of stress accumulations, sequential stressors, simultaneous stressor, habituation or compensation.

Finally, the model was developed in order to characterize the effect of an acute stress during life history of an animal. However, we assume that this type of model can be used at smaller or broader dimension scales, from cell to species by looking at shorter to longer time periods. Thanks to the genericity of the model, it becomes for example conceivable to apply the model to the effect of climate change perturbations on species fitness traits. A sudden change in environment is expected to lead to strong alterations of fitness traits like physiological performances and reproductive outputs [32,33]. In consequences, populations affected by this perturbation avoid extinction through adaptive plasticity or evolutionary adaptation. These two mechanisms lead to a shift in phenotypes bringing the fitness traits back to the values prior to the perturbation [34]. The rate and the capacity of adaptation throughout generations can be assessed using the model described in this study. Indeed, by fitting the model to longitudinal data on fitness values of species subjected to an environmental change can help extracting values of the sensitivity and the adaptive capacities of the species, two indispensable features to estimate the vulnerability of a species to climate change [35].

## Conclusions

The spring-damper model appears to be a good, simple, solution for modeling responses to an acute perturbation. It provides a new way to explicitly describe, analyze and compare responses of individuals facing an acute perturbation. The model fitted satisfactorily to physiological and behavioral responses to a confinement challenge in fish. Given that it also fitted responses to a nutritional challenge in goats, it seems likely that it can adjust to many types of biological system responses. This suggests that such physical models may be usefully applied to characterize robustness in biological systems.

## Supporting Information

S1 Appendix R code for the spring and damper model. (R)
